# Invasive mucinous adenocarcinoma: genetic insights into a lung cancer entity with distinct clinical behavior and genomic features

**DOI:** 10.1038/s41379-021-00945-0

**Published:** 2021-11-19

**Authors:** Reinhard Buettner

**Affiliations:** Institute for Pathology, University Hospital and Medical Faculty, University of Cologne, Kerpener Str. 62, D-50937 Cologne, Germany

**Keywords:** Cancer, Genetics research

**Fig. 1:**
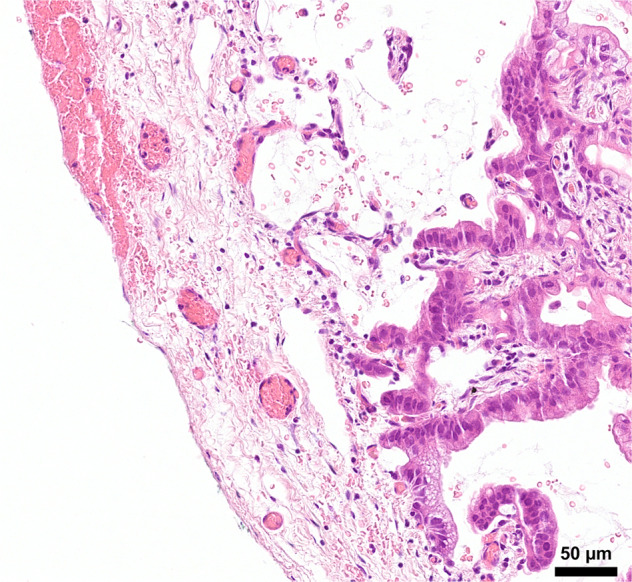
Histology of invasive mucinous adenocarcinoma (IMA) of the lung. This case shows both columnar and goblet cell differentiation, presented as a multifocal bilateral tumor and revealed a truncal *KRAS*G12D oncogenic driver mutation. The pleural surface is shown in the left side indicating the peripheral tumor location.

Invasive mucinous adenocarcinoma (IMA) is defined by the WHO classification as a primary lung adenocarcinoma with tumor cells showing goblet cell- or columnar cell-morphology (Fig. 1) with abundant intracytoplasmic mucin^1^. Due to its distinctive clinical features, i.e., peripheral location and a high frequency of multifocal, multilobular, and bilateral occurrence it has been defined as a distinctive entity with dismal outcome for many years and formerly been referred to as mucinous bronchioloalveolar carcinoma. Previous studies provided evidence for molecular features distinct from non-mucinous adenocarcinomas, with frequent *KRAS* mutations resembling RAS alterations in gastrointestinal tumors and oncogenic fusions in *KRAS* wild-type IMAs, as well as distinct clinical characteristics such as predominant recurrences in the lungs and a more aggressive phenotype for *NRG1*-rearranged tumors^2,3^.

Two manuscripts provide deep genomic insights and focus on the clonal relationship of multifocal IMAs using whole-exome sequencing^4^ and DNA-based next-generation sequencing of a large targeted gene panel^5^. Kim and colleagues from South Korea analyzed East Asian patients and report that, despite its multilocular presentation, IMAs share early and clonal initiating driver events, such as *KRAS*, *NKX2-1*, *TP53*, or *ARID1A* mutations. Overall tumor mutational burden was low, but still intratumoral heterogeneity was detected. Interestingly the predominant mutational signatures were signature 1 (endogenous mutational process), signature 2 and 13 (APOBEC activity), and signature 6 (DNA mismatch repair) but not related with smoking signature. Consistently, all patients were either never-smokers or had ceased smoking in their past history. This is somewhat surprising as IMAs are believed to be associated with demographic smoking habits, not different from non-mucinous lung adenocarcinomas^1^. The most frequent clonal oncogenic drivers in this study were activating *KRAS* mutations G12D, G12S, G12V, and Q61H. This is quite different from non-mucinous adenocarcinomas in East Asian patients where the most frequent drivers are activating mutations in *EGFR* exons 19 or 21^6^. Interestingly, the authors showed that multiple IMAs arising in the background of usual interstitial pneumonia (UIP) revealed distinct genomic features and hence represented multiple independent primary tumors. Thus, clonal multilocular and multiple primary IMAs in UIP seem to represent different origins of an otherwise morphologically similar tumor entity, mostly driven by clonal *KRAS* mutations. Further studies are needed to determine the risk of multiple IMAs in UIP.

Yang and colleagues from New York sequenced IMAS using the MSK-Assay “Integrated Mutation Profiling of Actionable Cancer Targets” for profiling more than 400 genes by DNA-based NGS and 62 genes for RNA-based NGS. Overall genomic analyses provided sufficient variants for calculating clonality versus non-clonality with significant confidence. All but one of 24 tumors revealed clonal *KRAS* G12D or G12V mutations and very few other driver alterations as a *F11R-NRG1* fusion, a non-canonical *BRAF*K483E mutation and an *ERBB2* exon20 insertion. Only three of their tumors revealed a *KRAS*G12C mutation. The NRG1 fusion is quite interesting as *NRG1* fusion events were originally identified from an IMA^7^. Thus, all oncogenic driver alterations appear to converge in a pathway of *EGFR/HER2* receptor activation or downstream alterations in the *KRAS/BRAF* signaling cascade. Smoking habits were not reported in detail, however, the overall smoking burden in this cohort was 13 pack years, indicating that smoking may occur in patients with IMAs but may not be the main driver of tumorigenesis. This data is also supported by previous studies reporting different genomic profiles between mucinous and non-mucinous adenocarcinomas and low association with smoking^2,3^. Interestingly, none of the patients revealed radiological signs of interstitial lung disease (ILD), and hence the two cohorts from Korea and the US may reflect different epidemiological backgrounds.

The most striking feature of both studies remains the strong difference in types of *KRAS* mutations between IMAs and non-mucinous adenocarcinomas. While in non-mucinous carcinomas, *KRAS*G12C is the most frequent driver with an incidence of almost 45%^8^, more than 90% of IMAs reveal KRAS G12D,V or other rare activating mutations. The mystery of entity-related KRAS mutations has been noted previously, for example as striking differences in KRAS mutation types between pancreatic, colonic, and non-mucinous lung adenocarcinomas. It is very likely that the establishment and outgrowth of adenocarcinomas may be dependent on different *KRAS* mutations under the control of specific homing factors. Obviously, G12C is not favored by the local milieu in the peripheral lung and further experimental mouse models studying the local tissue-specific requirements are needed to address this question. In any case, the successful clinical development of *KRAS*G12C-specific inhibitors^9,10^ will not change the dismal outcome of most patients with IMAs. Also, early detection is unlikely to prevent disease progression as early airborne spread frequently prevents local surgery. Thus, current development of pan*KRAS* inhibitors^11^ remains the cornerstone for possible future treatment options.

In summary, both studies by Kim (this issue) and Yang underline the importance of deep molecular profiling of all lung cancers currently summarized under the broad umbrella of “lung adenocarcinoma”. This umbrella comprises a broad spectrum of quite different tumor entities and molecular profiling will not only lead to more precise and effective therapies in advanced stages but also better diagnose the precise tumor entity with its specific risk profile for progression and need for adjuvant therapies in locally confined resected tumors^12^. Thus, pathologists need to integrate morphological features and genomic profiles for precision diagnostics in lung cancers both in advanced and in localized stages.
